# Overcoming challenges to access ecological function: a marine soft-bottom perspective

**DOI:** 10.1098/rsos.241176

**Published:** 2024-10-23

**Authors:** A. Martins, M. Di Domenico, Y. Costa, F. Barros

**Affiliations:** ^1^ Laboratório de Ecologia Bentônica, CIENAM & Instituto de Biologia & INCT Estudos Interdisciplinares e Transdisciplinares em Ecologia e Evolução, Universidade Federal da Bahia, Salvador, Bahia, Brazil; ^2^ Laboratório de Ecologia Marinha. Centro de Estudos do Mar, Universidade Federal do Paraná, Pontal do Paraná, Paraná, Brazil; ^3^ Centro de Ciências Agrárias, Ambientais e Biológicas, Universidade Federal do Recôncavo da Bahia, Cruz das Almas, Bahia, Brazil

**Keywords:** functional approach, functionality, macrobenthos, marine sediments, biodiversity

## Abstract

Ecosystem functioning studies have gained prominence due to concerns about the decline of species. In marine sediments, benthic invertebrates perform important ecological functions. However, the challenges within the field of functional ecology are sparsely discussed. Our aim was to systematize the problems and suggest pathways forward. To achieve this, we review recent articles on functional ecology on marine sediments and identified two main issues. First, the absence of a clear definition of terms. Second, in terms of applicability, we observed a mismatch between the scales of functioning and traits, incomplete information on trait databases, inappropriate selection of traits, subjective measures of traits, overlooked trait variability and data analyses without the link between the taxonomy and the traits associated with functions. We propose some pathways to overcome the challenges, such as (i) reasoning of the concept of function, process and trait, (ii) increasing experiments to measure functions, (iii) clarification of the relationship between traits and functions, (iv) clear procedure for assigning trait scores, (v) experiments for understanding trait variability and function performance, and (vi) analyses that consider the sets of traits of each taxon. Overcoming these challenges will allow us to advance research and fill gaps in knowledge of ecosystem functioning.

## 1. The functional approach in marine and coastal sediments

The interest in studying ecosystem functioning gained prominence in the last decades. This is mostly due to concerns about the consequences of the decline in biodiversity and the loss of species important to ecosystem functions and services [1–3]. The definition of ecological function varies depending on the scale and ecological system [4,5], and there is no consensus in the literature [1]. For example, at the ecosystem level, coral reefs’ functions may refer to the role of protecting shorelines due to their morphology and rigid structure, creating a barrier that reduces wave action. Estuaries function frequently as a vital feeding area for many species, including those commercially exploited (e.g. [6]). Nevertheless, at the community level, herbivorous reef fishes contribute to the control of algae growth, and estuarine polychaetes burrowing and tube building foster the oxygenation of marine sediments (e.g. [7–9]).

The analysis of specific traits, such as body size, feeding mode, mobility and reproductive traits, at the species level, forms the basis of the functional trait approach (e.g. [1,10]). This approach has been widely employed in studying ecosystem functioning [11–14] and biomonitoring [15,16]. A frequently used definition of functional traits is the characteristics of organisms that are relevant to their response to the environment and their impact on ecosystem functioning [17,18]. These traits can also indirectly affect fitness by influencing growth, reproduction and survival [18]. Nevertheless, it is well accepted that to establish the significance of a trait in ecosystem processes, such as its functional role, a comprehensive understanding of the organism and its interaction with the environment is necessary. This understanding enables the assessment of how the trait influences and contributes to ecosystem dynamics [19].

Marine and coastal ecosystems are highly valuable due to the multiple ecological services they provide, such as supporting fisheries, water quality, flood protection, climate regulation and recreational activities (e.g. [20–23]). Within these ecosystems, benthic organisms play crucial roles in marine soft bottoms, such as bioturbation, secondary production, organic matter fragmentation and sediment stabilization (e.g. [9]). These organisms influence the ecosystem through the functions they perform, affecting productivity rates, nutrient fluxes and carbon storage [8,24,25]. Studies have explored the relationships between benthic organism activities and environmental variables, including contaminants, salinity and granulometry (e.g. [26–32]). However, several challenges, such as inter/intraspecific trait variability, mismatch scale with trait, trait selection, measurement and analysis, and incomplete trait information search, persist in the field of benthic ecology.

In contrast to some areas, for instance, plant ecology, where significant methodological advances have enabled effective measurement and assessment of ecosystem functioning (e.g. [33,34]), several problems in benthic ecology remain largely unsolved. A delay in marine benthic function studies compared with terrestrial plant ecology can be attributed to several factors, including knowledge gaps, methodological challenges, taxonomic complexity and limited datasets. These challenges faced by benthic ecologists are overlooked and dispersed in the literature. Recent publications address the problems from a more critical perspective, but they have not clearly identified or systematically organized them (e.g. [35,36]) and/or focus on other taxonomic groups and environments [37]. Therefore, this study aimed to review and quantify these potential problems and suggest pathways for better understanding ecological function in soft sediment assemblages. By focusing on specific issues, this approach offers a more detailed perspective on the challenges and proposes practical solutions.

## 2. Methods

### 2.1. Review of problems

**Figure 1.**
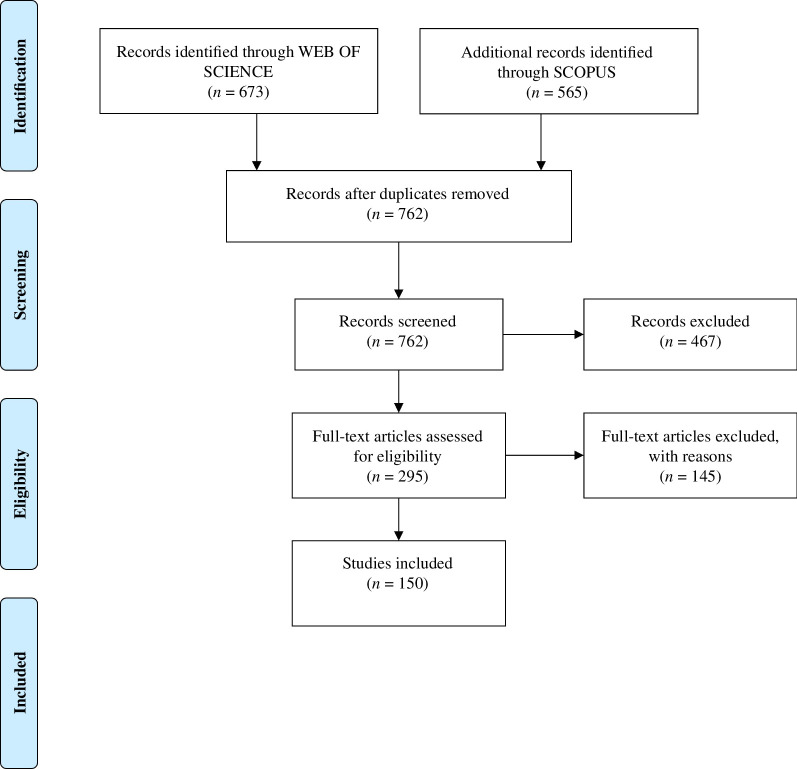
Flowchart of the article selection and review process in the research, adapted from PRISMA [39].

To systematize the problems, we sampled articles from the literature on the ecological functioning in marine and coastal environments. The databases Web of Science (WoS, Thompson Reuters; webofknowledge.com) and Scopus were used to search and compile all studies published before March 2023. The literature searches were performed using the combination of the words: ((Func*) AND (trait*) AND ((estuar* OR marine OR coastal) AND (benthic* OR invertebrate* OR macrobenthic* OR macroinvert*)) in the field of ‘topics’ (Article title, Abstract, Author, Keywords and Keywords plus). We used the review software Rayyan to process reviewing and managing the published literature [38]. After removing duplicates, this search resulted in 762 published studies considering the two search bases used. A pre-screening of the articles was performed using specific inclusion and exclusion criteria. First, we selected articles that aimed to relate benthic organisms traits with functioning, excluding articles focused on non-soft sediment benthic organisms (e.g. plankton, coral reefs and fishes) and that did not use trait analyses. Articles written in a language different from English were also excluded. We included articles that analysed the functioning of the marine ecosystem using macrofauna and meiofauna through traits (biological, functional or morphological). In the first screening, 295 articles were included after excluding duplicated studies and applying the exclusion criteria. Of this total, 150 articles focusing on the functioning of marine sediments were found in the last 5 years (2018–2023) (figure 1). To identify recent issues in the functional approach to marine benthic sediments, we focused on studies from 2018 to 2023. This 5-year span allows us to review current and persistent problems in this area. Additionally, we believe that earlier studies have probably addressed and solved many challenges. Moreover, there has been a significant increase in the number of publications on this topic in recent years, making it important to understand the current problems considering contemporary methodologies. This will help us develop strategies to overcome these challenges effectively. To assess the main problems, each of the 150 selected articles was carefully reviewed, and relevant information was recorded using Microsoft Excel software (electronic supplementary material, table S1). Information was registered regarding the definition of terminologies associated with traits and functions, measurement methods, analysis, taxonomic group, identification level, measured ecological functions, traits used and sources of trait information (electronic supplementary material, tables S1–S9). This information aided in quantifying and qualifying the encountered problems. To summarize the results, some frequency graphs were constructed using R software (R Core Team). A review of the terminology used to refer to the traits and functions was conducted in each of the selected articles. Subsequently, to visualize the most common terminologies used in functional ecology, a word cloud was constructed based on the frequency data of the terminologies per article, highlighting the main conceptual/terminological issues (electronic supplementary material, table S3). We also used a Sankey diagram to visualize the relationship between the measured functions, the most frequently used traits, and the types of analyses employed. Due to the large number of different trait nomenclatures used, we standardized and regrouped the categories of traits cited in the articles (for example, we grouped in category feeding all nomenclatures such as feeding strategy, trophic guild, diet type, feeding mode, feeding habit, trophic type and food type) (electronic supplementary material, table S4). The number of different analyses was also large, and we grouped similar analyses in multivariate statistical techniques used to explore relationships between variables and/or to reduce the dimensionality of data (i.e. nMDS, Bioenv, dbRDA, RDA, PCA, DISTLM, FCA and CA), analyses for group comparison and analysis of variance (i.e. PERMANOVA, ANOVA, ANCOVA, ANOSIM and SIMPER), regression models (GAM, GLM, linear model and mixed models) and mathematical models (differential equations and species distribution modelling). Moreover, we grouped indexes: biotic indices (AMBI, M-AMBI and BENTIX), functional diversity indexes (FRich, FEve, FDis, FDiv, FSpe and functional β) and other indexes (i.e. BIPc, BPc, BQI and IPc). Finally, the techniques were added to the other category, appearing only once in the review (i.e. meta-analysis, random forest, network analysis and hierarchical modelling of species).

## 3. Results and discussion

**Figure 2.**
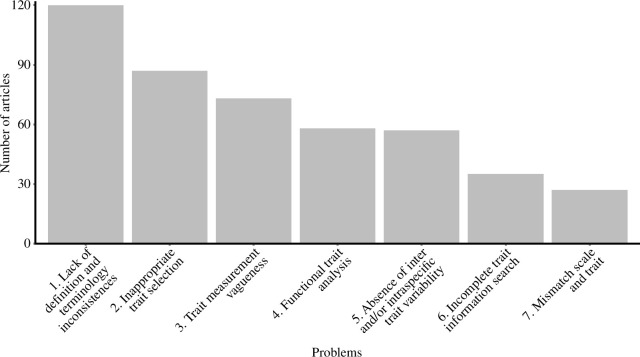
Frequency of problems associated with the functional approach in the articles. Conceptual problems (1) and applicability (2–7).

First, we observed that studies of ecological functions in soft sediments have problems of two different natures: (i) conceptual and (ii) related to the applicability of the approach (table 1). We observed that the central conceptual problem is the lack of explicitly presenting widely accepted and comprehensive definitions of ecological function, functioning, trait terminology and the use of terms (biological trait, functional trait) interchangeably within the same publication (figure 2; table 1, item 1.a). The applicability problems, on the other hand, range from the inappropriate selection of functional traits, mismatch of the scale, limitations in trait information in the databases, subjective measurement of traits, absence of inter- and/or intra-specific trait variability, and issues in the trait analysis (figure 2; table 1, items 2.b–g). We will discuss each of these issues in detail in the following sections.

**Table 1.** Synthesis of the main problems associated with the functional trait approach.

problem nature	problem	description	suggestions
1. conceptual	a. lack of definition of ecological function and terminological inconsistencies	missing definition of function, trait, and terminologies; lack of consistency in using the different terms	— explaining the used concepts of function and functional trait; — considering the response and effect of the trait (not only to about ‘the individual’s fitness’); — adopting terms in accordance with the definitions.
2. applicability of the approach	b. inappropriate selection of functional traits	mismatch selected traits with function; no traits selection; subjective selection, no justify	— selecting traits directly linked to the functioning; — considering theory for choosing traits; — *in situ* experiments to measure the effect of different traits on the environment; — avoid redundant traits.
	c. trait measurement vagueness	subjective measurement of traits; no fuzzy code explicitly	— justify the assignment of scores; — clear fuzzy coding procedures (so that study results can be used by other authors); — investing efforts *in situ* experiments; — proposing protocols for standardized measurement of functional traits.
	d. functional trait analysis issues	analyses that shuffle many traits of information from different species at once	— use analyses that consider taxon sets of traits rather than just multiple unitary traits independently of the taxon.
		comparing taxonomical versus functional diversity using species-based versus trait-based information	— to make the comparisons both components should have equivalent information.
		no trait analysis	
	e. absence of intra and/or interspecific trait variability	no consideration of inter- and/or intra-specific trait variability and environmental conditions	— invest in experiments that take into account the relationships of different species and individuals’ density in the ecosystem functionality.
	f. incomplete trait information search	limitation of traits information (e.g., absence of information for some taxonomic groups) trait data missing	— search more than one database; — wide literature search and expert consulting on taxa’s life history and ecology.
	g. mismatch scale and trait	recycling of traits under different study scales	— understand the functionality of traits under different scales; — clarity on the scale of your study.

### 3.1. Lack of definition and terminology inconsistencies

**Figure 3.**
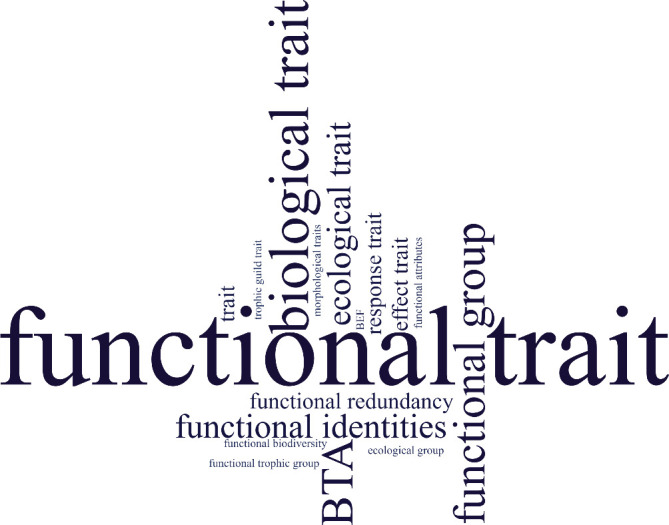
The word cloud of terminologies related to the functional approach obtained in the literature. The size of each word is proportional to the frequency that the word was used in all studies.

**Figure 4.**
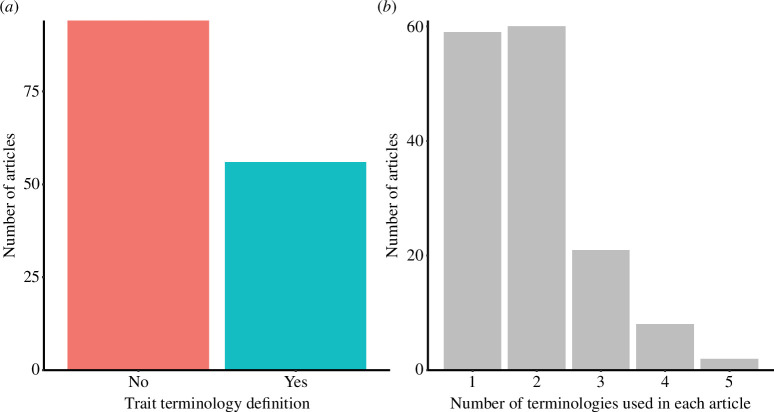
Frequency of studies that: (*a*) presented definitions of trait terminologies (biological trait, BTA, functional trait, functional group); (*b*) used one or more different terminologies in the same article.

The definitions of function, functioning and trait are often missing in the studies that investigate ecological functions in marine sediments (electronic supplementary material, table S1). Different trait terminologies have been used (e.g. functional trait, biological trait, effect trait and response trait) (figure 3) but are not clearly defined in many cases (electronic supplementary material, table S2 and figure 4*a*). Furthermore, there is a lack of consistency in the use of different terms, i.e. biological traits and functional traits are used interchangeably throughout the text (figure 4*b*). This seems to be a widespread problem in ecology. For example, in the study by Dawson *et al*. [40], which conducted a comprehensive survey on the use of the term ‘trait’ in ecology, the authors found that despite efforts to unify and clarify the terminology, the proposed definitions for traits were not consistently used or widely accepted by the ecological community. This indicates that, although there have been initiatives aimed at resolving these issues, the problem of inconsistent terminology persists. Anyway, there is no consensual definition in the literature, as also presented by Weiss & Ray [41], thus we propose that functional traits are characteristics of an organism that either have an impact on or respond to ecosystem dynamics and are intrinsically related to ecosystem processes essential for delivering ecosystem services [42–45]. On the other hand, biological traits encompass any measurable morphological, physiological or phenological characteristics at the individual level, ranging from cellular to whole organism levels, without reference to the environment or any other organizational level [12,18,46]. For instance, biological traits may include morphologies (such as morphological structures, the presence of eyes and body shape), but if they are not explicitly linked to functions in the environment, i.e. not necessarily functional. All traits of an organism are considered biological, indicating their potential significance for the organism’s fitness and performance [18]. However, not all traits are inherently functional, meaning they do not necessarily exert a significant influence on ecosystem processes depending on the context, scale and object to which they relate [19]. Due to the lack of a clear distinction between the definitions of biological and functional traits, functional diversity is typically regarded as a compilation of all traits available. It is important to, before labelling any biological trait or group of traits as functional, explicitly explain what ecological function it is performing or expected to perform. Simply labelling any biological trait as functional does not make any particular advance in understanding functioning. We suggest that studies must describe their concepts of function and use traits that match the research question. That is, for example, to consider burrowing behaviour as one effect (increasing sediment oxygen) trait as an influence that organisms can have on ecosystem processes and desiccation tolerance as a response trait, a possible improvement on the ability of a species to colonize or to persist in face of an environmental change.

The definition of terminologies associated with traits should be clear to the authors and guide trait selection. A recent review proposes some recommended definitions that can be used in aquatic trait-based studies and can guide the authors to maintain consistency across various trait terminologies [47]. If the desired function to be studied is clear, it is possible to consider the mechanisms that enable its performance in the environment and, consequently, the expected effects and/or responses. In this way, it is necessary to justify and explicitly describe the choice of traits. For example, sediment bioturbation is a function that enables the oxygenation of the lower sediment layers. Therefore, traits that allow organisms to access the lower layers and perform processes facilitating water entry and solute mixing, such as organism movement, are important to consider. Polluted environments may present anoxic sediments and will restrict organisms without traits capable of withstanding certain low oxygen conditions. In these conditions, traits such as burrow construction (which involves connecting to oxygen from the water column) and a physiological capacity for tolerating low oxygen levels are crucial. These traits offer valuable insights into the ecosystem’s functioning.

### 3.2. Inappropriate selection of functional trait

The absence of an explicit definition and disregard for theoretical responses and effects of the traits may lead to practical problems (table 1, item 2.b). The selection of traits and their explicit association with a specific function can be a great challenge due to the lack of information and direct measurements of the species. In our review, more than 60% (93 studies) did not justify trait choice and 116 studies (corresponding to 77% of the total) did not establish a link between trait and function (electronic supplementary material, table S1). Only 26 studies (17% of the total) utilized traits that were justified and directly linked to function. The chosen traits must be related to the studied functions, establishing a clear link between trait and function. For example, in sediment bioturbation, traits directly related to this function would include body size, movement mode, feeding mode and sediment compartment. That is, organisms with larger bodies capable of digging into the sediment at greater depths and feeding on the deposits will facilitate the ventilation of lower layers, thus bioturbating the sediments [48–51]. The ecological question must guide the selection of functional traits [52]. For instance, there is no logical justification to consider egg size (reproduction trait) when making comparisons of bioturbation between sites subject to different degrees of microplastic pollution.

Redundant traits may be selected in research studies and can result in misleading outcomes or numerical noise. For example, within the same study, both feeding mode and diet might be included, yet many of their individual categories exhibit strong overlap. For example, suspension feeding is frequently associated with a diet consisting of plankton and/or suspended particulate matter; carnivory aligns with an animal-based diet and deposit feeding aligns with a detritus-based diet [53]. Several approaches can be taken to avoid redundant traits. First, to conduct a specific review of the functional ecology of the studied taxa and systematize the most relevant and distinct traits associated with the functions or responses of interest. Additionally, consulting experts in the field of functional ecology of marine benthos will provide valuable insights on significant traits, avoiding redundancies. Furthermore, performing a correlation analysis among the considered traits enables the identification of highly correlated traits, which may indicate redundancy. Traits with no contribution must be discarded, and within redundant traits, a careful selection must be made. It is also crucial to consider the research question or hypothesis and identify traits that are most relevant and informative. These steps will improve the understanding of the functional ecology of marine benthos.

A recent study highlights the importance of standardizing trait nomenclature and provides a roadmap to help select traits based on their informativeness and availability [54]. This approach directly addresses the issue of trait selection by offering clear guidelines and reducing the ambiguity that often plagues trait-based studies. By following these guidelines, researchers can ensure that the chosen traits are both relevant and well-defined, improving the consistency and reliability of functional ecology research.

### 3.3. Trait measurement vagueness

**Figure 5.**
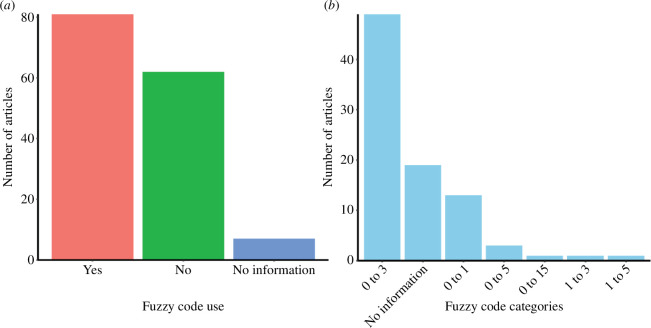
Frequency of studies (*a*) that use or do not fuzzy code approach and (*b*) the fuzzy code category applied in each study (0–3; 0–1; 0–5; 0–15; 1–3 and 1–5). No information refers to articles that did not use classification according to fuzzy logic and therefore do not have ranges of scores to count.

The trait information may be organized in different ways, from purely categorical classifications, continuous measures, or semi-quantitative approximations, such as ordinal categories or the use of fuzzy coding (i.e. scores are assigned to express the affinity of species with traits, e.g [55]; figure 5*a*). The absence of standardization in the measurement of functions is a problem that hampers comparisons of ecological functioning ([56]; table 1, item 2.c). This is because subjective trait scores (e.g. different intervals of scores 0–1; 0–3; 0–5; 0–15; 1–3 and 1–5) are given by different researchers to the same taxon for a given characteristic, often without presenting logical methodological steps (figure 5*b*). We need to standardize fuzzy coding, or at least justify it, allowing study results to be completely understood and the fuzzy code properly used or adjusted in future studies. In addition, if continuous traits are converted into categorical traits (e.g. body size), the authors should clarify the choice of classification adopted (table 1, item 2.c). For instance, the development of a handbook for plant trait measurement has resulted in a better balance between whole-plant traits, leaf traits, root and stem traits, and regenerative traits. It also places particular emphasis on traits that are important for predicting species’ effects on key ecosystem properties [33]. A similar approach is much needed for benthic ecology. Additionally, there is a need for greater clarity in translating biological traits into functional traits. Thus, theoretical tools are crucial for enabling a reasonable interpretation of results in ecological terms. For instance, Fontana *et al*. [57] provide a conceptual framework that proposes a continuum of trait integration. This continuum allows for a nuanced approach to integrating biological traits, which can aid in the creation of functional traits.

### 3.4. Functional trait analysis issues

**Figure 6.**
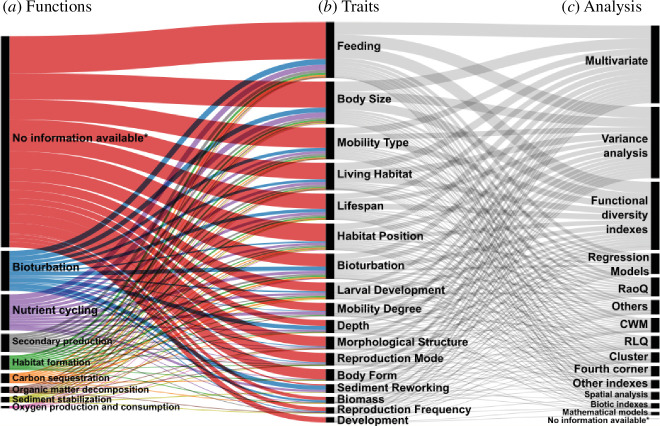
A Sankey diagram that provides a summary of the articles’ frequencies, connecting various ecological functions (*a*), traits (*b*), and types of analyses used (*c*) within each study. In cases where no specific information is provided (i.e. function, trait or analysis), it is denoted as ‘No information available*’. The traits used in more than 10 articles were represented in the diagram. For a detailed explanation of the grouped analysis, please refer to §2.

The majority of studies that propose analysis to ecosystem functioning using traits did not explicitly specify the function to which the sets of traits were related (figure 6). Furthermore, different analysis methods have been adopted (figure 6). The main studied functions through functional traits were bioturbation, nutrient cycling, secondary production, habitat formation, sediment stabilization, organic matter decomposition, carbon sequestration and oxygen production and consumption (figure 6). For example, the bioturbation function was assessed through the traits sediment reworking bioturbation mode, habitat position and feeding, mainly. The most used analyses were multivariate (e.g. nMDS, SIMPER and PCA), functional diversity indexes (e.g. FRich, FEve and FDiv) and variance analysis (e.g. PERMANOVA and ANOVA) Some more recent studies have used emerging machine learning approaches (e.g. random forest) [58], indicating that this is a research topic that presents a possibility for growth through the application of new analytical approaches.

The main problems associated with the adoption of different methods to analyse ecosystem functioning are (i) some indices mix multiple species traits to infer functioning and do not take into account the individual set of traits that each species must have for each function, and (ii) confounded comparisons between taxonomic and functional diversity (table 1, item 2.d). Often, studies compile any accessible trait and designate them as functional, to compare them with species richness using taxonomy. Some studies solely rely on species names to calculate taxonomic diversity and compare it with the diversity of functional traits. But if there are many more variations in species names than in the number of traits (or vice versa) it will result in flawed comparisons. This issue falls under the same problem of making comparisons between studies using different taxonomic resolutions (e.g. comparing species with family level). To make equivalent comparisons, the trait information for both components should be used (i.e. morphological traits for taxonomic diversity and functional traits to functional diversity analysis) [19]. For this, the traits used to quantify functional diversity should consider traits that are functional, meaning they are relevant to an organism’s response to the environment and/or its effects on ecosystem functioning. In addition, if we need to assess the effect of species on functioning, use analysis that considers a species set of traits independently. Additionally, it is necessary that before data analyses, it is necessary to construct a matrix containing the taxon traits, the functions and the link with the sets of traits to perform them. Finally, we will then have access to the functions that each taxon is capable of performing. Another point is that each trait, or combination of traits, does not perform the function with the same intensity. For example, a polychaete that is tubicolous, very motile and large performs more bioturbation than a polychaete that is tubicolous, sedentary and small. For this, it is important to assign scores based on the affinity of each trait, or subsets of traits, with a given function, and a maximum score is assigned to the combination that best performs each function [9].

### 3.5. Absence of inter- and/or intra-specific trait variability

Species can play different functional roles depending on the biotic and abiotic settings [59]. For example, animal–sediment interactions can result in different effects across environmental gradients, resulting in habitat-dependent effects (e.g. crabs burrowing in muddy or sandy sediments can have very different effects) [60]. Studies have identified that species of polychaete spionids exhibit different feeding behaviours, such as consuming suspended or deposited particles, and increasing their feeding rate depending on the water flow and presence of currents [61,62]. However, inter- and/or intra-specific trait plasticity and potential changes due to environmental conditions have not been considered in many ecological functioning studies (table 1, item 2.e). The bioturbation potential (BPc), which incorporates abundance, biomass, mobility and sediment reworking with fixed factors in an equation, falls in capturing the contextual or spatio-temporal variations and the interactions between different species [51]. For example, species can exhibit varying bioturbation behaviours depending on their reproductive stage, but this aspect is not considered in these equations. It is important to recognize that trait analyses often provide ‘potential’ estimates of functioning, as individual organisms may not always exhibit the complete potential of their species’ traits. However, potential intraspecific variability in trait values cannot be disregarded. For instance, alterations in species’ behaviour in response to external stimuli or context-specific adjustments (site with different granulometry) to reworking and mobility traits, should be appropriately considered in scores. This contrasts with the assumption that an organism’s functional effects and responses will be consistent within and between populations. To fully access ecosystem function, we need to invest in the refinement of information regarding of ecological roles of key species, species-environment interactions and inter- and intra-specific relationships (i.e. sets of traits and density of traits).

### 3.6. Incomplete trait information search

Another frequently observed problem is the diffuse and lack of information on the functional trait sources (e.g. databases, literature). For instance, while the WORMS database provides some trait information, it could enhance the knowledge available for benthic species. It is important to note that some trait information generally is influenced both by the used taxonomic level (e.g. species or genus) and that the availability of trait information is regionally variable (e.g. global north greater than global south; temperate greater than tropical) (table 1, item 2.f). Consolidated ecological knowledge at the species level is generally available for a restricted and well-investigated species pool, frequently higher for those most common and widespread species [63].

In our review, we classified the studies based on their nature (manipulative or non-manipulative) and observed that the lack of trait information may be related to the scarcity of manipulative studies focused on determining species traits. Only a small percentage (4%) of the studies were manipulative (conducted *in situ* or the laboratory) and successfully measured ecological functions while establishing a link between functions and relevant traits (electronic supplementary material, table S1). Additionally, we emphasize the need for manipulative experiments to test the relationships between traits and specific ecological functions so that we can gather evidence leading to a more objective selection and a better understanding of the mechanisms through which species influence their environment, rather than selecting traits that do not have a direct relationship with the function in question. For example, we have very little information on how much organic matter a polychaete omnivore with armed jaws can fragment per cubic centimetre per hour or how much oxygen a tubiculous polychaete provides for a few centimetres in the sediment per day.

To overcome this challenge, it is necessary to invest in basic research (i.e. taxonomical and ecological experiments) to collect relevant data on trait information for specific taxonomic resolutions. Trait searches must be performed in more than one reliable database, and that information is compared. In addition, it is necessary to access the literature and specialists on the life history and ecology of the focal taxa. We believe that an updated consolidated database should unify all functional trait information, instead of several different ones. For plant ecology, a plant trait database TRY is successful and potentially a model for trait database initiatives [34]. For marine regions, the functional trait database FUN Azores has compiled information on meio-, macro- and mega-fauna. However, there are still gaps in information regarding traits relevant to ecosystem recovery (e.g. reproductive type, larval development, growth rates and lifespan) [64]. In the context of marine benthic sediments, there are recent examples of initiatives that have compiled trait information for many taxa and provide online access [65]. However, these initiatives are still focused on regions in Europe and at the genus level. It is necessary to extend this information to different regions around the globe and refine it to the species level. We suggest that a benthic trait database should (i) involve systematic extraction of trait data from public repositories, published literature and input from specialists in life histories of benthic organisms, (ii) implement a data integration (like the approach used in plant trait databases) and keep the system up to date, and (iii) involve collaboration among countries and institutions. This would benefit the scientific community and promote global biodiversity conservation efforts.

### 3.7. Mismatch scale and trait

Processes operating at different scales may have different effects depending on the response traits considered in the analysis. There must be a match in the scale of the investigated function and the selected functional traits. For instance, studies that examine ecological function at a large scale (i.e. regional scale; electronic supplementary material, table S2) often fail when they do not consider traits that vary across these scales, such as juvenile dispersal, recolonization [66]. Caution must be taken whenever using traits originally used by studies with a different scale (table 1, item 2.g). The scales at which experiments are conducted can diverge from the scale at which ecosystem functions change [67]. We need to focus on strategies to link traits with functions at the individual level and with ecosystem functions. Studies should consider that the functioning of marine benthos can vary with different environmental settings and scales. Spatial scale influences the environmental context to which these organisms are subjected, affecting how factors such as granulometry, salinity, concentration of organic matter and dissolved oxygen are distributed and interact. For example, sandy beaches can be classified into macro-, meso- and micro-scales. The macrobenthic community shows variations in the body size depending on differences in grain size and wave exposure in a macroscale pattern among beaches and variations in mobility depending on the availability of organic matter and food on a microscale within the beaches [68]. Investigating this variation will provide valuable insights into how ecosystems respond to different conditions and can help ecological modelling to simulate different scenarios. So, understanding the functionality of traits under different scales and assuming the potential limitations is essential to approximate ecosystem functioning in the real world. Some pathways that can be explored in different research projects are studying traits at the level of individual organisms, populations, communities and ecosystems, investigating trait–environment relationships, implementing monitoring programmes to track trait functionality over time and different scales, developing ecological models that incorporate trait-based approaches and account for scale-dependent effects, trying to integrate data from more diverse sources such field observations, genetic studies and ecological surveys, conducting controlled experimental studies. Recent studies have focused on mapping ecological changes on a larger scale by connecting small-scale experiments with broader landscapes through interdisciplinary approaches. For instance, the integration of optical imaging and computer vision techniques will enable both quantitative and qualitative assessments of biodiversity, the diversity of functional traits and ecosystem functioning [36]. These strategies will provide a comprehensive understanding of trait functionality, helping overcome scale limitations.

## 4. The future of ecological functional approaches in marine soft-bottom sediments

The discussed problems influence the interpretation of results and make it difficult to compare studies on functional ecology, predict the impacts of future environmental variations on ecosystem functioning and develop conservation strategies. In contrast to recent studies that highlighted the limitations of the functional approach in the benthos [35], we strongly emphasize the importance of proper experimental work for advancing the field of ecological function. We showed that few studies have established a direct correlation between the selected features and their corresponding functions. Such connections were primarily observed in manipulative experiments, where the effect or response of the employed traits was explicitly described. For example, large-scale field experiments manipulate nutrient concentrations along environmental gradients to test trait responses [69]. So, experimental work is necessary and can inform about ecological problems [70].

Continued integration of the functional approach with different disciplines (chemical, physical and geochemical) will improve the understanding of the mechanisms of eutrophication, dredging, oil contamination, sea-level rise and lost habitat can change the distribution of benthic organisms and the associated ecological functions. Studies in the southern Baltic Sea have demonstrated that oxygen depletion significantly impacts the functional diversity of benthic macrofauna, limiting their ecological roles and reducing resilience to environmental stressors [71].

The synthesis and discussion of these problems are a crucial step towards overcoming challenges associated with the functional approach. The contribution of this study is in quantifying and describing the problems of the functional approach in marine ecosystems. This helps us to understand how each of the problems might influence one another. For example, thelack of a clear definition of traits and functions is a central issue, affecting the selection of traits based on the authors’ understanding of their relevance to the function.

We detail all the problems, providing real data from reviewed studies in the field which is not extensively covered in other studies (e.g. [72]). This study explicitly explains how each function relates to traits and analyses, emphasizing the importance of selecting traits directly linked to the function. Understanding which specific functions have limited information to link with traits helps focus on techniques and experiments to reduce subjectivity in this association.

By identifying specific aspects of each issue, this study summarizes these problems, contributing to developing practical solutions and advancing our understanding of marine sediment functioning. From there, we can conduct research that aims to fill the gaps in our knowledge about ecosystem functioning. The problems mentioned here are interconnected, and ignoring any of them will hinder our progress.

**Figure 7.**
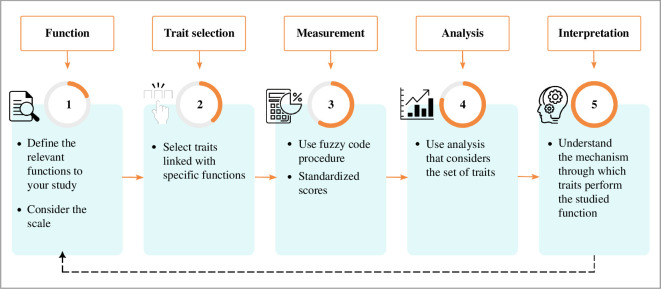
A roadmap to investigate function using a functional trait approach. The dashed line indicates the return to the interpretation of the function.

Therefore, we suggest five essential steps to consider when conducting studies using functional traits to assess function (figure 7). First, identify the function of interest to be studied and its relevance to the ecosystem and the taxa studied and have a clear understanding of the scale that pertains to this function, whether it is at the individual or ecosystem level. Second, select functional traits applicable to benthic species of your interest (morphological, physiological and phenological) that are important in the context of the focal benthic ecosystem. Third, use transparent, consistent and standardized approaches which will enable a clear understanding of the data analysis and interpretation. Fourth, analyse and describe the analyses carefully having in mind the function. Fifth, interpret the results, in the light of the relationship between species traits and ecosystem function, and discuss their implications for benthic ecosystem ecology. Collaboration with experts in the field is crucial for high-quality and relevant research, for which it is important to share results with other benthic trait ecologists, participate in discussions to validate results, and ensure robustness.

## Data Availability

Dataset: [73]. Supplementary material is available online [74].
